# Impact of a psychiatry clerkship on stigma, attitudes towards psychiatry, and psychiatry as a career choice

**DOI:** 10.1186/s12909-015-0307-4

**Published:** 2015-03-07

**Authors:** Zaza Lyons, Aleksandar Janca

**Affiliations:** School of Psychiatry and Clinical Neurosciences, M521, The University of Western Australia, 35 Stirling Highway, Crawley, Perth, 6009 Western Australia Australia

**Keywords:** Medical students, Stigma, Career, Attitudes, Psychiatry, Clerkship

## Abstract

**Background:**

Mental illnesses are a major public health problem around the world and the prevalence and burden of common mental disorders is growing. Psychiatry is an unpopular career choice for many medical students and this impacts negatively on the supply of psychiatrists to the workforce. The psychiatry clerkship can play an important role in influencing students’ attitudes towards psychiatry, either positively or negatively. However, stigma towards mental illness detracts students from considering a career in psychiatry. The aim of this study was to assess the impact of an eight week psychiatry clerkship on i) student knowledge and interest in psychiatry; ii) psychiatry as a career choice; iii) attitudes towards psychiatry; and iv) perceptions of stigma towards mental illness.

**Method:**

Year 4 medical students at the University of Western Australia completed two questionnaires, the Balon Attitudes Towards Psychiatry and the Mental Illness Clinicians Attitudes (MICA), at the beginning and end of the psychiatry clerkship. Interest in, knowledge of, and consideration of psychiatry as a career were also assessed. Non-parametric tests were used to compare baseline and follow-up differences on the Balon and MICA. Unpaired t-tests compared mean differences for interest, knowledge and psychiatry as a career.

**Results:**

Attitudes towards psychiatry were positive at the beginning of the clerkship. Overall, there was a significant decrease in negative and stigmatising views towards mental illness post clerkship measured by the MICA, but the follow-up mean score remained close to the neutral value with views in some areas becoming more negative. There was no significant improvement in students’ interest in psychiatry post clerkship, however, knowledge of psychiatry improved significantly. Numbers of students ‘definitely considering’ psychiatry as a career increased significantly from 7 (4.6%) students at baseline to 17 (10.5%) at follow-up.

**Conclusion:**

The clerkship made a modest impact on students’ attitudes to psychiatry, stigma and consideration of psychiatry as a career. Integration of strategies to overcome stigma towards mental illness and the mental health profession into pre-clinical teaching may provide students with skills to prepare them for the clerkship. This may assist in improving attitudes towards psychiatry and encourage more students towards a psychiatry career.

## Background

Mental illnesses are a major public health problem around the world [1]. They are common, disabling and debilitating conditions [2] that impact negatively on a person’s quality of life, their ability to lead productive and fulfilling lives and form meaningful family, social and personal relationships [3]. Furthermore, people with mental illness are among the most stigmatised, marginalised and vulnerable members of society and suffer from discrimination in many areas of daily life as a consequence of their illness [4]. The prevalence and incidence of common mental disorders are predicted to rise in the coming decades. In particular, age related conditions including dementia will increase and add significantly to the overall global burden of disease caused by mental illness [5,6].

In order to ensure that the psychiatric workforce remains sustainable into the future, new generations of motivated and enthusiastic young doctors need to be encouraged towards a career in psychiatry. However, a recent systematic review concluded that while medical students attitudes towards psychiatry are generally positive, psychiatry as a potential career choice is unpopular [7]. A comprehensive international survey of medical students in 20 countries found that overall, only 4.5% of students were ‘definitely considering’ psychiatry as a career [8]. As a result of this ambivalence towards psychiatry, recruitment to postgraduate psychiatry training has been consistently low for decades. Analysis of the career choices of newly qualified doctors in the UK found that from 1974 to 2009 psychiatry was the first career choice for around 3-5% of medical graduates per year [9] and similar trends have been reported in other countries [10]. In its 2012 report, Health Workforce Australia reported a current shortage of psychiatrists, and estimated that by 2025 this would worsen significantly and result in a shortage of up to 452 psychiatrists, one of the highest levels of shortage across all areas of medical specialisation [11].

Stigma towards mental illness has increasingly been identified as a factor that influences medical student attitudes towards psychiatry and detracts them from considering psychiatry as a career [12-14]. Medical students often have stigmatised views towards mental illness prior to commencement of their medical training [15,16]. Negative views can be further strengthened when students start clinical clerkships in psychiatry and have contact with mentally ill patients [12,17]. Clerkship studies have found that students feel uncomfortable with patients [18], feel that mentally ill patients have a poor prognosis [19] and that interacting with patients is stressful [20]. In other studies, students have reported that working with patients is dangerous [21], disturbing, emotionally draining and overwhelming [15,22].

Medical students considering psychiatry as a career will often be subject to stigmatising comments regarding their choice from others, including family members and friends [15,23], further alienating them from psychiatry as a career. For psychiatrists, stigma often persists throughout their career [10] with the profession perceived as having a negative image, both in the community and by other medical specialists [24,25].

Clerkships (also called attachments, placements or rotations) form a core component of clinical teaching in medical schools. Psychiatry clerkships are often the first exposure that students have to patients with mental illness, psychiatric wards and mental health services more generally. A U.S. study found that the length of the psychiatry clerkship varied between medical schools ranging from 4–8 weeks, averaging around 6.2 weeks [26]. A systematic review of the impact of the psychiatry clerkship that assessed 26 studies from 19 countries found that the average length of clerkship was 5.5 weeks with 4 weeks the most common length [27]. Clerkships provide a good opportunity for academics and others involved in clinical teaching to promote psychiatry to students, including its career potential. However, while they have a positive effect on students attitudes towards psychiatry, there is mixed evidence of their impact on psychiatry as a career choice [27].

In order to investigate this apparent disparity between positive attitudes towards psychiatry as a discipline and negative attitudes towards psychiatry as a career, and explore the role that stigma may play, a survey of medical students was undertaken. The aim of the study was to assess the impact of the eight week clinical clerkship in psychiatry on i) student knowledge and interest in psychiatry; ii) psychiatry as a career choice; iii) attitudes towards psychiatry; and iv) perceptions of stigma towards mental illness.

This paper will report on the results of this study and discuss strategies that may assist to improve students attitudes towards mental illness and psychiatry as a career choice.

## Method

### The psychiatry clerkship

The current medical course at the University of Western Australia (UWA) is a six year undergraduate Bachelor of Medicine, Bachelor of Surgery (MBBS) degree. During Year 4 students rotate through four different eight week clinical clerkships, including psychiatry, in groups of approximately 60. These clerkships are the first opportunity that students have to experience working in hospitals and other clinical settings. For the psychiatry clerkship students are divided into smaller groups of 4–8 and allocated to a clinical teaching site that is attached to a psychiatric inpatient unit. During the eight weeks they have several additional shorter visits to old-age and alcohol and drug rehabilitation services. As well as ward work with a clinical team including consultants and registrars, students also have a three hour tutorial per week which is facilitated by an academic staff member and covers theoretical components of psychiatry. These tutorials cover a range of case based learning scenarios including mood disorders, anxiety disorders, schizophrenia, substance abuse, personality disorders and organic disorders. A two day introductory course of lectures is given at the beginning of the clerkship. The main component of assessment is a case presentation where students are required to interview a patient for an hour followed by a presentation of the findings and discussion with the examiner.

### Study design and procedures

Study participants were Year 4 MBBS medical students at UWA who were undertaking their eight week psychiatry clerkship. Students were asked to complete two questionnaires on the first day of the clerkship (baseline) and again towards the end of the clerkship (follow-up). Questionnaires were distributed during tutorial contact time and participation was voluntary. To ensure confidentiality no identifying information was collected.

Ethics approval for the study was granted from the UWA Human Research Ethics Committee. Consent to participate in the survey was implied if students decided to complete the survey.

The Balon Attitudes Towards Psychiatry questionnaire [14] and the Mental Illness Clinicians Attitudes Scale (MICA) (medical student version) [28] were used in the study. The Balon questionnaire was developed in 1999 and has been used in a number of studies that have been conducted in different countries to measure medical students attitudes toward psychiatry. It has 29 items and is rated on a 4 point scale – strongly agree, moderately agree, moderately disagree, strongly disagree. The following themes are covered: i) overall merits of psychiatry; ii) efficacy; iii) role definition and functioning of psychiatrists; iv) possible abuse and social criticism; v) career and personal reward; and vi) specific medical school factors.

The medical student version of the MICA was developed and validated in 2010 [28]. It has 16 items and is rated on a 6 point Likert scale – strongly agree, agree, somewhat agree, somewhat disagree, disagree, strongly disagree. The minimum total score is 16, maximum 96. A lower score indicates a less stigmatising attitude towards mental illness and psychiatry.

In addition to the Balon and MICA, demographic data was collected. Students were also asked to rate their interest in, and knowledge of psychiatry on a 10 point visual analogue scale (1 = low interest/knowledge; 10 = high interest/knowledge) and their extent of consideration of psychiatry as a career on a 10 point scale (1 = definitely not considering; 10 = definitely considering).

Statistical analyses were carried out using IBM SPSS software, Version 22.0. Unpaired t-tests were used to compare mean differences on the visual analogue scale questions. Non-parametric tests were used to compare baseline and follow-up differences on Balon and MICA items. As the full range of responses on the 4 point Balon rating scale had not been utilised by many respondents on a number of the questions, the rating scale was dichotomised into ‘agree’ and disagree’ variables and the percentage agreement/disagreement for each item was calculated. McNemars test was used to determine significance between baseline and follow-up on each item. For the MICA, the negatively worded questions were reverse scored and the mean total baseline and post scores calculated. An unpaired t-test was used to determine statistical significance on the mean scores, a Mann–Whitney test tested differences between male and female students and a Wilcoxon signed rank test determined any significant changes between baseline and follow-up on each item. The significance level for all statistical testing was set as p = <0.05.

## Results

Approximately 238 students were invited to participate in the baseline survey, and 230 to the follow-up survey. One hundred and fifty one students responded to the baseline survey (63% response rate), and 161 responded to the follow-up survey (70% response rate). At baseline, 68 (45%) respondents were male and 83 (55%) female. At follow-up 77 (48%) were male and 84 (52%) female. The mean age was 23 years, range 20–40 years.

### Interest, knowledge and psychiatry as a career

The baseline means for interest and knowledge of psychiatry, and psychiatry as a career were 5.7/10, 3.5/10, and 3.9/10 respectively. The follow-up means for interest and knowledge of psychiatry, and psychiatry as a career were 6.1/10, 6.0/10, and 4.8/10 respectively. An unpaired t-test found that there were no significant changes at follow-up on the level of interest in psychiatry t(310) = 1.6133, p = 0.1077. There was, however, a significant improvement on the level of knowledge of psychiatry, and interest in psychiatry as a career, t(310) = 15.4053, p = 0.0001 and t(310) = 3.3804, p = 0.0008 respectively. Details are shown in Table 1.

**Table 1:** **Changes in interest, knowledge and psychiatry as a career**

	Baseline mean ( *sd* )	Follow-up mean ( *sd* )	*t*	p value
Interest in psychiatry	5.7 (1.8)	6.1 (2.0)	1.6133	0.1077
Knowledge of psychiatry	3.5 (1.6)	6.0 (1.3)	15.4053	0.0001
Psychiatry as a career	3.9 (2.0)	4.8 (2.2)	3.3804	0.0008

Mann–Whitney U tests found that they were no significant differences between male and female students at either baseline or follow-up in interest in psychiatry (baseline p = 0.123; follow-up p = 0.394), knowledge of psychiatry (baseline p = 0.240; follow-up p = 0.663) or psychiatry as a career (baseline p = 0.756; follow-up p = 0.451).

The number of students definitely considering a career in psychiatry (those who scored the question as 8, 9, or 10/10) rose from 7 (4.6%) students at baseline to 17 (10.5%) at follow-up.

### Attitudes towards psychiatry

Overall, students reported positive attitudes towards psychiatry, both at baseline and follow-up. Only three Balon items showed a statistically significant change post clerkship, all of which were in the positive direction. These were, ‘I feel uncomfortable with mentally ill patients’; ‘Teaching at my medical school is interesting and of good quality’; and, ‘Although I am interested in psychiatry, no effort was made to encourage my becoming a psychiatrist at my medical school’. Items that assessed the overall merits of psychiatry and the efficacy of psychiatry were all favourably rated. Eighty-seven percent at baseline and 91% at follow-up agreed that, ‘Psychiatry is a rapidly expanding frontier of medicine’ and 84% at baseline and 81% at follow-up disagreed with the statement, ‘Psychiatry is unscientific and imprecise’.

With one exception, the items that measured the role, definition and functioning of psychiatrists were all positively rated at baseline, with little capacity for significant improvement at follow-up. One item, ‘Among mental health professionals, psychiatrists have the most authority and influence’ showed a more divergent viewpoint. Agreement at baseline was 59%, increasing to 68% at follow-up, however, this was not significant.

Students’ were positive about the teaching of psychiatry during the rotation. Seventy-five percent agreed at baseline that teaching was of a good quality and this increased significantly to 93% at follow-up, McNemars p = 0.001. Approximately 90% reported that residents, registrars and consultants they met during the rotation were good role models. There was significant improvement in students views on the level of encouragement to pursue psychiatry as a career, 60% disagreed at baseline with the statement, ‘Although I am interested in psychiatry, no effort was made to encourage my becoming a psychiatrist at my medical school’ compared with 77% at follow-up, McNemars p = 0.001.

Several of the ‘career and personal reward’ items showed less positive attitudes, specifically those that assessed how students and others perceive psychiatry as a discipline and career. Fifty-two percent at baseline agreed that psychiatry has a low prestige among the public and there was no significant change in this at follow-up (47%). Approximately a third of students at baseline agreed that their family and friends would discourage them from a career in psychiatry and there was no change in this at follow-up. There was only a 22% agreement with, ‘Psychiatry has a high status among other medical disciplines’ dropping to 17% at follow-up, but the difference was not significant.

The percentage agreement/disagreement relating to each item and McNemars test are shown in Table 2 below.

**Table 2:** Balon **attitudes towards psychiatry – baseline and follow-up** agreement, and significance

	Baseline	Follow-up	McNemars
	*Agree (%)*	*Agree (%)*	
	*Disagree (%)*	*Disagree (%)*	
**Overall merits of psychiatry**			
1. Psychiatric research has made good strides in advancing care of the major mental disorders	94	96	NS
	6	4	
2. Psychiatry is a rapidly expanding frontier of medicine	87	91	NS
	13	9	
3. Psychiatry is unscientific and imprecise	16	19	NS
	84	81	
**Efficacy**			
4. If someone in my family was very emotionally upset and the situation did not seem to be improving, I would recommend a psychiatric consultation	83	91	NS
	17	9	
5. Psychiatric consultation for medical or surgical patients is often helpful	90	93	NS
	10	7	
6. Psychiatric treatment is helpful to most people who receive it	91	91	NS
	9	9	
**Role, definition and functioning of Psychiatrists**			
7. Psychiatry is not a genuine and valid branch of medicine	5	4	NS
	95	96	
8. Most psychiatrists are clear, logical thinkers	93	94	NS
	7	6	
9. With few exceptions, clinical psychologists and social workers are just as qualified as psychiatrists to diagnose and treat emotionally disturbed persons	22	28	NS
	78	72	
10. Among mental health professionals, psychiatrists have the most authority and influence	59	68	NS
	41	32	
11. Psychiatrists are too frequently apologetic when teaching psychiatry	13	7	NS
	87	93	
12. Psychiatry is too ‘biologically’ minded and not attentive enough to the patient’s personal life and psychological problems	7	9	NS
	93	91	
13. Psychiatry is too analytical, theoretical, and psychodynamic, and not attentive enough to patient’s physiology	15	14	NS
	85	86	
**Possible abuse and social criticism**			
14. Psychiatrists frequently abuse their legal power to hospitalise patients against their will	3	5	NS
	97	95	
15. On average, psychiatrists make as much money as most other doctors	64	68	NS
	36	32	
**Career and personal reward**			
16. Psychiatry has a low prestige among the general public	52	47	NS
	48	53	
17. Psychiatry has a high status among other medical disciplines	22	17	NS
	78	83	
18. Many people who could not obtain a residency position in other specialities eventually enter psychiatry	15	17	NS
	85	83	
19. Psychiatry is a discipline filled with international medical graduates whose skills are of low quality	5	9	NS
	95	91	
20. My family would discourage me from entering psychiatry	32	37	NS
	68	63	
21. Friends and fellow students would discourage me from entering psychiatry	28	35	NS
	72	65	
22. If a student expresses interest in psychiatry, he or she risks being associated with a group of other would-be psychiatrists who are often seen by others as odd, peculiar or neurotic	30	30	NS
	70	70	
23. I feel uncomfortable with mentally ill patients	44	16	0.001
	56	84	
**Specific medical school factors**			
24. Teaching of psychiatry at my medical school is interesting and of good quality	75	93	0.001
	25	7	
25. During my psychiatry rotation, psychiatry residents were good role models	N/A	89	N/A
	N/A	11	
26. Attending psychiatrists during my psychiatry rotation were good role models	N/A	92	N/A
	N/A	8	
27. Most psychiatrists at my medical school are clear, logical thinkers	93	98	NS
	7	2	
28. Most non-psychiatry staff at my medical school are respectful of psychiatry	80	85	NS
	20	15	
29. Although I am interested in psychiatry, no effort was made to encourage my becoming a psychiatrist at my medical school	40	23	0.001
	60	77	

### Mental illness stigma

The baseline mean total score for the MICA was 48.2 (sd 8.3) and the follow-up mean total score was 43.5 (sd 7.5). An unpaired t-test found that this was a significant change, t(310) = 15.4053, p = 0.0001 indicating an overall improvement in attitudes towards psychiatry and mental illness stigma post clerkship. The baseline mean score for male students was 49.4 (sd 9.1) and 47.2 (sd 7.4) for females. A Mann–Whitney test found no significant differences between males and females at baseline (p = 0.068). However, at follow-up, the mean score for males was 42.0 (sd 6.0), and 44.8 (sd 8.4) for females, Mann–Whitney (p = 0.015).

The median scores and level of agreement/disagreement for each MICA item are shown in Table 3. Baseline and follow-up comparison of each MICA item was undertaken using Wilcoxon signed rank tests. Five items showed a significant change post clerkship. Two of these, ‘I feel as comfortable talking to a person with a mental illness as I do those with physical illness’; and ‘It is important that any doctor supporting a person with a mental illness also assesses their physical health’ showed a significant improvement in attitudes post clerkship. Three showed a more negative attitude post clerkship. These were, ‘People with a severe mental illness can never recover enough to have a good quality of life’; ‘Psychiatry is just as scientific as other field of medicine’; and ‘The public does not need to be protected from people with a severe mental illness’. Refer to Table 3 for details.

**Table 3:** **MICA** ^**1**^ **baseline and follow-up median disagreement and significance**

	Baseline	Follow-up	Baseline (%)	Follow-up (%)	Wilcoxon
	(Median)	(Median)	SA; A; SWA;	SA; A; SWA;	
			SWD; D; SD	SWD; D; SD	
Q1. I just learn about psychiatry because it’s in the exam and would not bother reading additional material on it	4	4	1; 2.5; 30;	2; 7.5; 17;	NS
			24; 32; 10.5	38.5; 24; 11	
Q2. People with a severe mental illness can never recover enough to have a good quality of life	5	4.5	1; 3; 5;	3; 6.5; 11;	0.001**
			26.5; 40.5; 24	29; 38; 12.5	
Q3. Psychiatry is just as scientific as other field of medicine	3	3	6; 42; 35;	7; 35.5; 27;	0.046**
			13; 3; 1	21; 8.5; 1	
Q4. If I had a mental illness I would never admit this to any of my friends for fear of being treated differently	3	4	4; 13; 39;	2.5; 9; 32;	NS
			18; 20; 6	28.5; 23; 5	
Q5. People with a severe mental illness are dangerous more often than not	5	5	0; 4.5; 15;	0; 6.0; 14.5;	NS
			29; 40; 11.5	29; 41; 9.5	
Q6. Psychiatrists know more about the lives of people treated for a mental illness than do family members of friends	3	3	2; 16; 44.5;	4.5; 15; 34;	NS
			24.5; 11; 2	28; 17; 1.5	
Q7. If I had a mental illness I would never admit this to any of my colleagues for fear of being treated differently	3	3	8; 23; 42;	9.5; 20.5; 37.5;	NS
			18; 7; 2	20; 10.5; 2	
Q8. Being a psychiatrist is not like being a real doctor	5	5	0; 2; 10;	1; 1; 8.5;	NS
			18; 14; 28	21; 41.5; 27	
Q9.If a psychiatrist asked me to treat people with a mental illness in a disrespectful manner, I would not follow their instructions	2	2	36; 42; 15;	39; 40.5; 11.5;	NS
			2.5; 2.5; 2	4.5; 2; 2.5	
Q10. I feel as comfortable talking to a person with a mental illness as I do those with physical illness	4	3	4.5; 18; 23;	12.5; 36; 27;	0.0001*
			38.5; 15; 1	19; 4.5; 1	
Q11. It is important that any doctor supporting a person with a mental illness also assesses their physical health	2	1	31.5; 53.5; 14;	52; 40.5; 6.5;	0.0001*
			0; 1; 0	1; 0; 0	
Q12. The public does not need to be protected from people with a severe mental illness	4	4	1; 11; 24.5;	2; 10; 20;	0.041**
			43; 19; 12	34; 22.5; 11.5	
Q13. If a person with a mental illness complained of physical symptoms, I would attribute it to their mental illness	5	5	0; 1; 7;	0; 2; 6.5;	NS
			33; 49; 10	34.5; 41; 16	
Q14. GP’s should not be expected to complete assessment for people with psychiatric symptoms as they can be referred to a psychiatrist	5	5	0; 2; 11;	1.5; 3; 7;	NS
			29; 46; 12	32.5; 42; 14	
Q15. I would use the terms ‘crazy’, ‘nutter’, ‘mad’ etc. to describe people with a mental illness who I have seen in my work	5	5	1.5; 4.5; 11;	1.5; 7; 15.5;	NS
			18; 35; 30	23; 26.5; 26.5	
Q16. If a colleague told me they had a mental illness I would still want to work with them	2	2	21; 57; 18;	31.5; 48.5; 15;	NS
			2; 1; 1	3.5; 1.5; 0	

^1^MICA – Mental Illness Clinicians Attitudes scale SA = strongly agree (1); A = agree (2); SWA = somewhat agree (3); SWD = somewhat disagree (4); D = disagree (5); SD = strongly disagree (6).

*significance demonstrates less stigma post clerkship; **significance demonstrates more stigma post clerkship.

## Discussion

This study explored the impact of an 8 week psychiatry clerkship on medical students’ attitudes towards psychiatry and mental illness stigma. Several different measures assessed interest and knowledge of psychiatry; attitudes towards psychiatry; and perceptions of stigma, both towards mental illness, people with mental illness, the discipline of psychiatry, and psychiatry as a career.

Knowledge and interest in psychiatry were poorly rated at the beginning of the clerkship. At the end of the clerkship there was no significant change in the level of interest in psychiatry, however, there was a significant improvement in students’ knowledge, which indicates that despite low interest in psychiatry, the teaching of the clerkship which was highly rated, resulted in an increase in knowledge.

Attitudes towards psychiatry measured using the Balon questionnaire showed that a number of items were positively rated at baseline, leaving little capacity for significant improvements at follow-up. Similar findings have been reported in other studies [29-32]. In particular, attitudes towards mentally ill patients improved significantly post clerkship and there was a correlation between similarly worded items on the Balon and MICA questionnaires, demonstrating a level of internal consistency between the two instruments. Improvements in attitudes towards patients post clerkship have been found in other studies [33,34] supporting the contact theory which proposes that contact with people with mental illness improves attitudes and acceptance towards mental illness [35]. There was also agreement between instruments regarding the scientific basis of psychiatry, which was positively rated on both the Balon and MICA, however, the MICA detected a small but significant negative change in this post clerkship.

The baseline and follow-up means for the MICA were close to the neutral value, (48.2 and 43.5 respectively). Despite a significant improvement at follow-up, this shows only weak evidence that the clerkship decreased stigma towards mental illness. The items regarding recovery of people with mental illness and the protection of the public from people with mental illness were more negatively rated post clerkship, and this has also been observed in other studies [19,36]. This could reflect the clerkship structure which exposes students to patients with more severe symptoms in acute public inpatient settings, where recovery is not captured or witnessed over the clerkship duration. The post clerkship improvement in students feeling comfortable talking to people with mental illness may be due to psychiatric history taking and assessment skills learned during the clerkship which resulted in increased confidence in interviewing and assessing patients. However, despite this improvement, the disturbed behaviours of patients observed in an acute setting may be interpreted by students as dangerous and could account for the view that the public need to be protected. Changes to the current clerkship structure to provide opportunities for students to work with patients in outpatient and community settings may provide a more realistic view of mental illness enabling students to see positive aspects of treatment and management.

The clerkship, which includes ward work with patient contact, and the weekly tutorial sessions decreased students’ perceptions of stigma to some extent, however, there is room for improvement. Addressing stigma through educational interventions is essential for psychiatry to overcome its negative status among students [23], but evidence of the effectiveness of anti-stigma training is mixed. Friedrich and colleagues found that an educational package of lectures, personal testimonies and role play activities improved short term knowledge, attitudes and behaviour, but these effects were not maintained over time [37]. A similar training package found improvements in knowledge, but not in attitudes and behaviour [38]. In order to maintain the positive benefits of anti-stigma training over a longer period of time, these strategies need to be embedded and integrated throughout the curriculum.

It was encouraging that the quality of the psychiatry teaching during the clerkship was highly rated and psychiatrists regarded as good role models. There was a significant increase in the consideration of psychiatry as a career which resulted in an additional 10 students who were ‘definitely considering’ psychiatry as a career post clerkship. Evidence of the impact of the clerkship on career preference is mixed, with some studies finding increases in the level of career interest post clerkship [34,39-41] and others finding no differences [32,33,42]. The clerkship structure, length of clerkship and specific cultural factors may account for the differences in career interest found in these studies that have been conducted in medical schools internationally. While attitudes post clerkship have been found to deteriorate over time as students continue with their studies and internship [43], a positive clerkship experience can have an enduring and positive effect if interested students receive ongoing encouragement from consultants, registrars and other academic staff members [44] as they progress through their training. This is particularly important as career decisions regarding specialisation in general are often made up to three years after graduation from medical school [25].

In recent decades, lifestyle factors have been more closely considered by medical students and junior doctors when making career decisions. These include maintaining a work-life balance; choosing a career with a family friendly image; having the opportunity to work part-time; and a manageable, controllable and flexible workload [15,18,20]. Psychiatry is well placed to provide positive lifestyle factors for its potential professionals [45], yet it remains an unpopular career choice. Negativity expressed towards psychiatry among friends, family, the public and other medical specialists, psychiatry’s poor social prestige and lower earning potential found in this, and other studies [14,20,46,47] may explain why some students do not regard psychiatry favorably as a potential career. Stigma on this level may exert a greater influence over career decision making processes and act to negate the more positive aspects that the discipline has to offer.

In order to mitigate these views, a further role of the clerkship could be to identify students who have a particular interest in psychiatry and provide them with support and mentorship to ensure continued interest over the remainder of the medical course and into internship [18]. Enrichment programmes such as Summer Schools, Psychiatry Institutes and electives can play an important part in targeting students interested in psychiatry and provide them with a more in depth educational exposure to the speciality [48-51]. These types of programmes have been successful in enhancing career interest in psychiatry and in psychiatry as a discipline and could be implemented by medical schools more widely. In addition, the encouragement of student led mental health interest groups also have the potential to encourage positive views towards psychiatry among students, destigmatise mental illness and demystify psychiatry [8].

### Study strengths and limitations

While a number of studies have identified stigma as an issue [12,17], our study was strengthened by the use of a relatively new instrument to measure stigma, the MICA. This adds a new dimension to what is known about the role that stigma plays on students attitudes towards psychiatry and to psychiatry as a career choice.

Limitations of the study are that in order to maintain confidentiality it was not possible to match baseline and follow-up responses as identifying information was not provided by respondents. For students’, providing identifying information in surveys is perceived negatively as, despite assurances by staff, they are concerned that their responses, in particular negative responses, may influence their assessment results or treatment by clinical and academic staff in some way. It can also impact negatively on the response rate. For these reasons, it was decided not to use a matched design which would have resulted in a more robust study design but compromised response rates and biased responses to some of the items in the questionnaires, particularly those relating to the treatment of patients and quality of teaching. Not all students in the year group participated in the study and it is possible that selection bias towards those students who are more interested in psychiatry may have contributed towards the improvement in attitudes reported in the results.

This 8 week clerkship takes place in the fourth year of a six year medical course and is the first real exposure that students have to the practical aspects of working with patients with mental illness. At the end of the clerkship there were improvements in attitudes and overall perceptions of mental illness stigma, however, while we can conclude that the clerkship may have contributed towards this improvement, we cannot assume that these views will remain constant as students’ progress through the course. There is no certainty that the students at follow-up who stated that they were ‘definitely considering’ psychiatry will go on to pursue it as a career. Longer term follow-up of the same cohort of students will enable attitudes to be tracked over time to determine how to maintain the gains made during the clerkship and develop appropriate teaching resources that will provide opportunities to further improve attitudes and stigma.

The clerkship is comprised of a number of different components including ward work and tutorials. Future research in this area that specifically assesses each component to find out what works best from a student perspective will help psychiatric educators to structure clerkships that address both the required and relevant educational outcomes and maximise opportunities to improve attitudes towards psychiatry, reduce stigma and increase the number of students who are considering psychiatry as a career.

## Conclusion

The discipline of psychiatry must be able to recruit and retain a viable workforce of psychiatrists that will adequately meet the future needs of the profession. This clerkship made a modest impact on students’ attitudes to psychiatry, stigma and consideration of psychiatry as a career. The integration of strategies to overcome stigma, both towards people with mental illness and the mental health profession, into pre-clinical teaching may provide students with skills to better prepare them for the negative aspects of the clerkship and could assist in improving attitudes towards psychiatry and encourage more students to consider psychiatry as a career. Finally, psychiatry is an integral part of the practice of medicine and it is important that, regardless of future area of specialisation, all students foster a positive attitude towards mental illness in order to provide holistic treatment for their patients.
